# Standardization of medium composition and agricultural waste in the production of *p*-hydroxybenzoic acid by *Paecilomyces variotii*

**DOI:** 10.1007/s13205-014-0262-5

**Published:** 2014-10-31

**Authors:** Jyothi Ramesh Jain, Jimcy Thalakootoor John, Ghosh Jyotirmoy, Shiragambi Hanmatagouda Manohar

**Affiliations:** 1 Department of Biotechnology, C.P.G.S. Jain University, 3rd Block, Jayanagar, Bangalore, 560011 India; 2 BCL, Biocon Research Limited, Biocon, Bangalore, 560100 India; 3 National Institute of Animal Nutrition and Physiology, Adugodi, Bangalore, 560030 India

**Keywords:** Biotransformation, Corncob, *p*-Coumaric acid, *p*-Hydroxybenzoic acid, HPLC

## Abstract

Biotransformation of *p*-coumaric acid by *Paecilomyces variotii* resulted in the accumulation of a metabolite which was identified as *p*-hydroxybenzoic acid. We studied the possibility of enhancing this metabolite by various parameters such as varying the concentration of substrate, addition of different nitrogen sources and carbon sources at varying concentration and use of different agricultural wastes as a nutritive medium. Maximum amount of 254.6 ± 9.34 mg/kg of *p*-hydroxybenzoic acid was detected in the solid medium of corncob on 4th day of incubation using 10 mM *p*-coumaric acid as a sole carbon source. Consequently, *P. variotii* can be used as candidate fungi for the production of *p*-hydroxybenzoic acid. Solid wastes can be managed by using them for biotransformation of pharmaceutically important phenolic acids and hence cut down the cost of production of value-added compounds using a low-cost medium.

**Electronic supplementary material:**

The online version of this article (doi:10.1007/s13205-014-0262-5) contains supplementary material, which is available to authorized users.

## Introduction

Phenolic acids and its derivatives exist in all plants and plant-based foods such as vegetables, fruits and grains. The major fraction is generally linked through ester or ether bonds with other components. Phenolic acids possess antioxidant (Cotelle 2001), anti-tumor (Rocha et al. 2012), anti-diabetic and anti-inflammatory responses (Araujo and Leon 2001) that makes them pharmaceutically important group of compounds (Robbins 2003). *p*-Coumaric acid is one of the major components of plant cell wall and is abundantly available in nature (Harris and Hartley 1980). Decomposition of *p*-coumaric acid is likely to occur during extraction using most commonly used methods such as acid hydrolysis and saponification. Enzymatic processes can be a safe alternative and recently there has been an increasing interest in using microorganisms for their extraction or transformation of phenolic acids (Stalikas 2007). The most common phenolic acid is *p*-coumaric acid and, therefore, it can be utilized as a substrate for the production of value-added phenolic acids such as *p*-hydroxybenzoic acid (Sachan et al. 2006).

Consequently, solid waste management is an alarming problem encountered by many of the developing countries across the globe. 140 billion metric tons of biomass is generated globally every year from agriculture sector (Kaur et al. 2013). This enormous amount of biomass can be converted into energy for household and industrial uses. A parallel set of industries has to be established that can utilize the agricultural waste to convert them into useful products for the sustainable management of the waste and for the sustainable economic growth of the country.

Phenolic acids are released as a breakdown product of lignin in plant cell wall and there has been a considerable interest in producing these phenolic acids from natural substrates (Reinoso et al. 2006). Raw materials and by-products of agriculture can be utilized to produce pharmaceutically important phenolic acids. The use of solid substrates can result in high yields and has received increasing scientific interest than any other methods. In this study, we report the biotransformation of *p*-coumaric acid into *p*-hydroxybenzoic acid by *Paecilomyces variotii* MTCC 6581 in suspension culture as well as using various agricultural wastes as a nutritive medium.

This kind of biotransformation of phenolic acids is important for the global carbon cycle from an environmental point of view as they are released as a breakdown product from plants (Peng et al. 2003). Earlier reports have shown that *Streptomyces, P. variotii* and *Pycnoporus cinnabarinus* can metabolize *p*-coumaric acid and produce *p*-hydroxybenzoic acid (Nambudiri and Bhat 1972; Alvarado et al. 2001; Sachan et al. 2005,  2006). Catabolic route of *p*-coumaric acid in white rot fungus *Schizophyllum commune* was also investigated (Sachan et al. 2010). Agricultural wastes do contain phenolic acids and there is a need for processing and extracting these phenolic acids to obtain value-added compounds.

## Materials and methods

### Culture, chemicals and agricultural waste

A pure fungal culture of *P. variotii* was obtained from MTCC (Microbial Type Culture Collection, Chandigarh; MTCC 6581) and was maintained on Potato dextrose agar slants at 37 °C for 7 days (Sachan et al. 2005). *p*-Coumaric acid and *p*-hydroxybenzoic acid standards were obtained from Sigma and the rest of the chemicals were procured from Himedia (India). Wheat bran, rice bran, sugarcane bagasse, corncob, paddy straw, peanut skin, pomegranate peel and wheat straw were obtained fresh from the field and or industries.

### Medium, culture conditions and extraction

Minimal media was used for the conversion of *p*-coumaric acid. Fungal spore suspension was inoculated into minimal media containing basal inorganic salts (Muheim and Lerch 1999). All the carbon sources were filter sterilized before their addition into the medium. 1 ml of fungal spore suspension was added into the flasks containing 25 ml of the medium supplemented with *p*-coumaric acid as a sole source of carbon and incubated up to 10 days. The culture filtrate obtained after filtration is acidified with concentrated hydrochloric acid to adjust the pH to 1–2. It was then extracted with ethyl acetate thrice and the organic layer was separated and dried. The residue left was dissolved in 50 % methanol and used to detect the biotransformed products (Sachan et al. 2006).

## Standardization of culture conditions

### Substrate concentration

Effect of various concentrations of *p*-coumaric acid on *p*-hydroxybenzoic acid formation was examined. Fungal spore suspension was inoculated on minimal media containing varying concentrations of *p*-coumaric acid (1.0, 2.5, 5.0, 7.5, 10.0 mM). The cultures were incubated for 10 days at 37 °C. The samples (three flasks 25 ml) were then harvested at an interval of 2 days and quantified (Sarangi and Sahoo 2009).

### Nitrogen sources

Fungal spore suspension was allowed to grow on minimal medium containing different nitrogen sources (ammonium nitrate, ammonium sulfate, potassium nitrate and sodium nitrate) and 10 mM *p*-coumaric acid as a carbon source. Analysis was carried out by sampling the cultures at an interval of 2 days for duration of 10 days.

### Carbon sources

Different carbon sources (glucose, fructose and sucrose) of varying concentration (0.25, 0.50, 1.00, 1.50 and 2.00 % w/v) was supplemented into the minimal medium (Sachan et al. 2005) and incubated for 10 days. Sampling of the cultures was performed at an interval of 2 days for a period of 10 days.

### Effect of agricultural waste

Different agricultural wastes (wheat bran, rice bran, sugarcane bagasse, corncob, paddy straw, peanut skin, pomegranate peel and wheat straw) were collected, dried and powered (140 US Mesh sized filter). Fungal spore culture was allowed to grow on 10 g of the raw materials taken in each of the conical flasks containing 10 mM *p*-coumaric acid as a carbon source to which 20 ml of distilled water was added (Bello et al. 2012). The culture was incubated at 37 °C for 10 days followed by addition of 30 ml of distilled water and subjected to extraction (Sachan et al. 2005, 2006). The biotransformed products were detected by analytical procedures––TLC and HPLC (Sachan et al. 2006; Sarangi and Sahoo 2009).

### Identification and quantification

Thin layer chromatography was performed for the identification of the biotransformed phenolic acid. The TLC analysis was performed as described by Pifferi (1965) and the samples that showed maximum intensity were quantified using HPLC (Figs. S1, S2, S3, S4). Quantification of the biotransformed products was performed on a Waters dual absorbance HPLC unit, equipped with a C_18_ column (4 µm, 3.9 × 75 mm) and the detector set at 254 and 310 nm. An isocratic linear solvent system of 1 mM trifluroacetic acid (72 %) and methanol (28 %), with a flow rate of 1.0 ml/min for 15 min at room temperature was used to elute the phenolic compounds. The phenolic compounds were identified by comparing it with the retention time of the standards and quantified by measuring the peak areas (Sachan et al. 2006). All the experiments were performed in triplicates. Statistical analysis was performed and a *p* value of <0.05 was considered significant.

## Results and discussion

### Effect of *p*-coumaric acid concentration

**Fig. 1:**
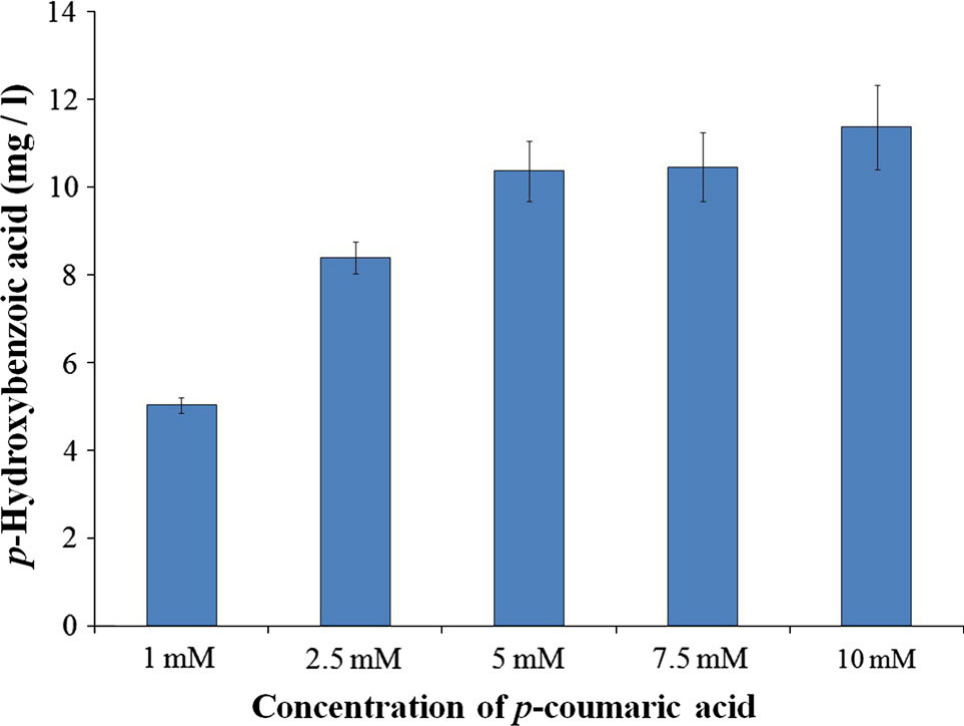
Effect of *p*-coumaric acid concentration on the production of *p*-hydroxybenzoic acid

*Paecilomyces variotii* was incubated in minimal media containing increasing concentrations of *p*-coumaric acid (1.0, 2.5, 5.0, 7.5 and 10.0 mM) as a sole source of carbon. Time course study of *p*-coumaric acid utilization was carried out and it was found that *P. variotii* was capable of transforming *p*-coumaric acid into *p*-hydroxybenzoic acid. It was observed that as the concentration of *p*-coumaric acid increased, there was an increase in production of *p*-hydroxybenzoic acid. A maximum of 11.36 ± 0.95 mg/l of *p*-hydroxybenzoic acid production was observed in the medium supplemented with 10 mM of *p*-coumaric acid on 8th day of incubation (Fig. 1). The slight variation was observed in the conversion rate of 5 and 7.5 mM of *p*-coumaric acid i.e., 10.36 ± 0.68 and 10.46 ± 0.77 mg/l and there is no explanation for the same.

Recently, a number of studies have been conducted to produce such pharmaceutically important compounds using various microbes as the focus remains on antioxidant and anticancer properties of hydroxycinnamic acids (Rahouti et al. 1989; Alvardo et al. 2003; Sachan et al. 2006). It was observed that higher concentration of substrate increased the production of *p*-hydroxybenzoic acid (Sachan et al. 2006). Sachan et al. (2006) observed the production of 200 mg/l of *p*-hydroxybenzoic acid and the production in the present investigation was very much lower (11.36 mg/l). This may be due to the effect of subsequent subculture of the organisms on the medium, due to decrease in its enzyme activity (Ekinci et al. 2006). **[**The *P. variotii* was isolated by Ghosh et al. (2006) from a dried mesocarp of tender coconut and was deposited at MTCC. By the time we procured, the culture would have undergone many subcultures and the process of cryo-preservation affects the productivity].

### Effect of nitrogen source

**Fig. 2:**
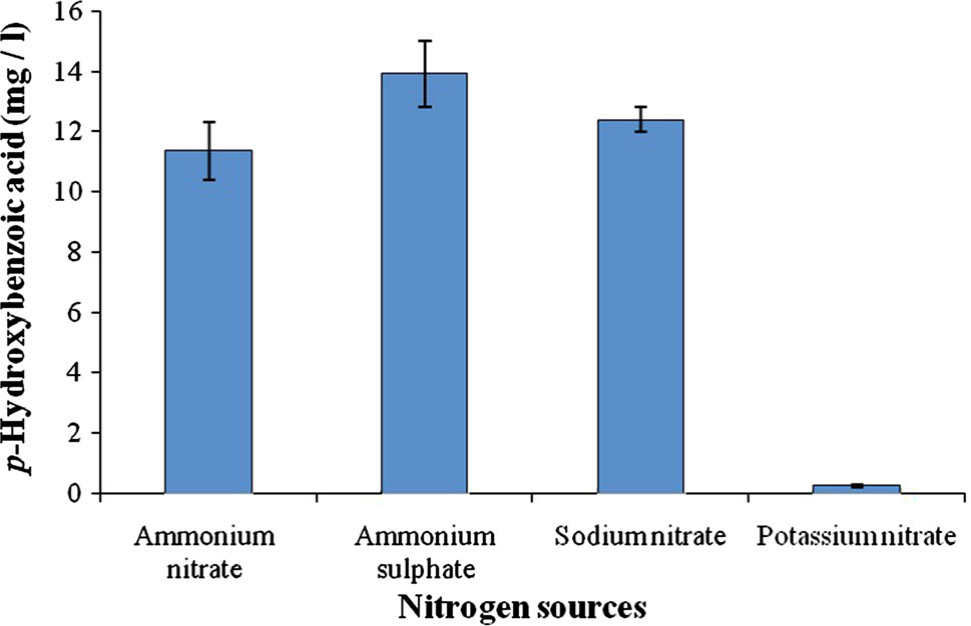
Effect of different nitrogen sources on the production of *p*-hydroxybenzoic acid from *p*-coumaric acid

Effect of various nitrogen sources on the production of *p*-hydroxybenzoic acid was investigated. It was found that ammonium sulfate could elevate the production to 13.9 ± 0.97 mg/l on 8th day of incubation as compared to ammonium nitrate present in the minimal medium. The production ranged from 11.3 ± 0.95 to 13.9 ± 0.97 mg/l when different nitrogen sources were used but the presence of potassium nitrate had a negative effect on the production of *p*-hydroxybenzoic acid (Fig. 2). Hence, it is necessary to choose suitable nitrogen source for production of phenolic compounds.

Nitrogen sources play an important role in the survival and reproduction of the organism. It is the main composition of DNA, protein and acts as a building block of the cells. Recently, in the biotransformation studies of meloxicam by *Cunninghamella blakesleeana*, the nitrogen source was varied and it was found that ammonium nitrate was most suitable for efficient production (Prasad et al. 2011).

### Effect of carbohydrate concentration

**Fig. 3:**
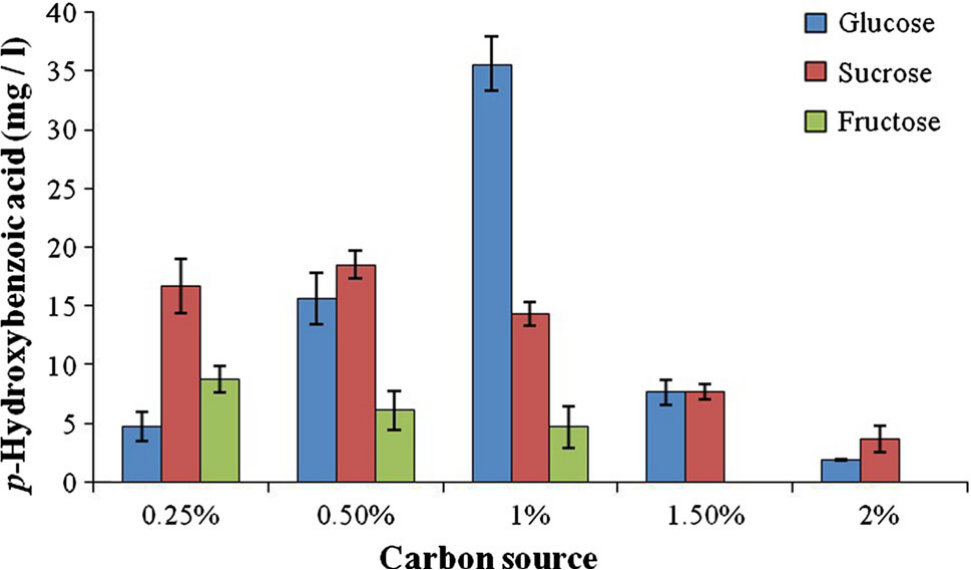
Effect of different carbon sources at varying concentrations on the production of *p*-hydroxybenzoic acid

Glucose, sucrose and fructose at varying concentrations (0.25, 0.50, 1.00, 1.50 and 2.00 % w/v) were supplemented into the medium along with 10 mM of *p*-coumaric acid and it was observed that 1 % glucose showed a better production with a maximum of 35.5 ± 2.28 mg/l *p*-hydroxybenzoic acid on 8th day of incubation (Fig. 3). The concentration of *p*-hydroxybenzoic acid decreased as the glucose concentration increased (other metabolites were not monitored), as the cell prefers to use the easily available carbon source when compared to complex *p*-coumaric acid. The presence of sucrose at 0.5 % was able to produce 18.49 ± 1.18 mg/l of *p*-hydroxybenzoic acid, and an increase in the sucrose concentration decreased the production (Fig. 3). The presence of fructose had a deleterious effect on the production of *p*-hydroxybenzoic acid as the concentration increased.

Glucose is the most preferred carbohydrate in fermentative processes and, therefore, addition of glucose at a particular concentration can help in the increased activity of the organism and thus elevated production (Prasad et al. 2011). Also, supplementation of different carbon sources can have various effects on the morphology, biomass and production of metabolites in fungus (Jia et al. 2009). We report for the first time the use of different carbon sources at varying concentrations for the transformation of *p*-coumaric acid into *p*-hydroxybenzoic acid.

### Effect of agricultural wastes on the production of *p*-hydroxybenzoic acid

**Fig. 4:**
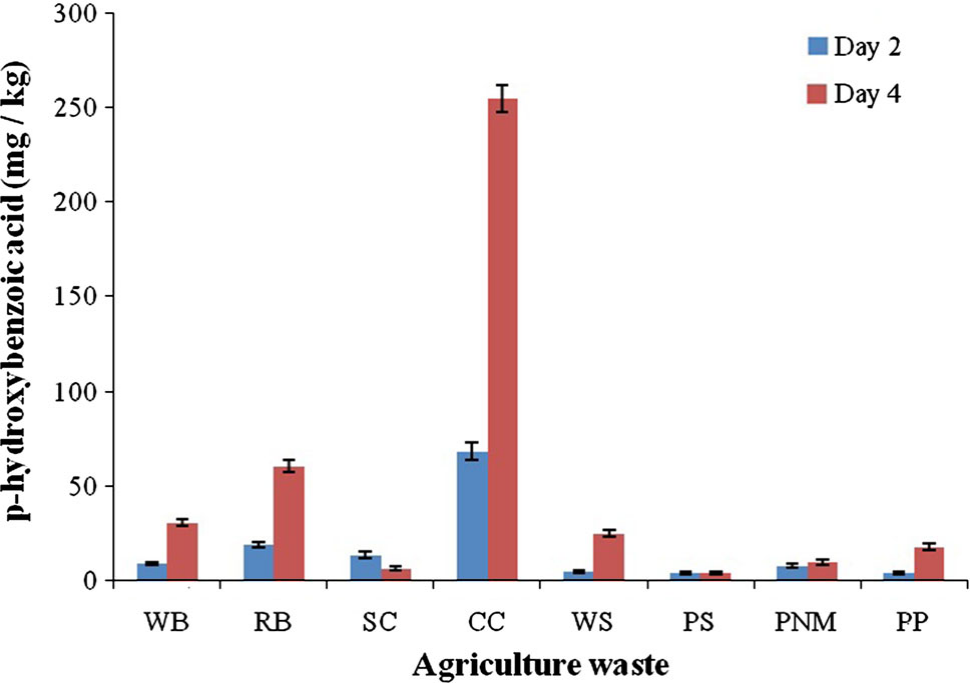
Effect of agricultural waste on the production of *p*-hydroxybenzoic acid (*WB* wheat bran, *RB* rice bran, *SC* sugarcane bagasse, *CC* corncob, *WS* wheat straw, *PS* paddy straw, *PNM* peanut meal, *PP* pomegranate peel)

Different agricultural wastes (wheat bran, rice bran, sugarcane bagasse, corncob, paddy straw, peanut skin, pomegranate peel and wheat straw) were used as a nutrient source for the production of *p*-hydroxybenzoic acid using *P. variotii*. The production of *p*-hydroxybenzoic acid was analyzed at various intervals of incubation time (2 and 4 days) and it was observed that corncob was able to produce a maximum of 254.6 ± 9.34 mg/kg of *p*-hydroxybenzoic acid on 4th day of incubation, followed by rice and wheat bran (60.54 ± 3.72 and 30.65 ± 3.37 mg/kg, respectively, Fig. 4). The production of *p*-hydroxybenzoic acid from other agricultural waste was too low compared with that of corncob, rice bran and wheat bran. Agricultural waste (corncob) was most efficient in the biotransformation of *p*-coumaric acid to *p*-hydroxybenzoic acid as compared to the minimal media and, hence, these solid wastes in a way can be utilized commercially for producing value-added compounds.

## Conclusion

The filamentous fungus, *Paecilomyces variotii*, appears to be a promising organism for the production of *p*-hydroxybenzoic acid from *p*-coumaric acid. This study demonstrates the use of nutrient supplements and various agricultural wastes for production of the potent antioxidant and anti-cancer compound, *p*-hydroxybenzoic acid. The use of agricultural waste (corncob) is of great potential for production of value-added compounds having pharmaceutical importance, hence solving a problem for waste management as well.

## Electronic supplementary material

Below is the link to the electronic supplementary material.

Supplementary material 1 (JPEG 39 kb)

Supplementary material 2 (JPEG 45 kb)

Supplementary material 3 (JPEG 40 kb)

Supplementary material 4 (JPEG 13 kb)

Supplementary material 5 (DOC 13 kb)
