# Supplementary figures and images for: Standardization of medium composition and agricultural waste in the production of p-hydroxybenzoic acid by Paecilomyces variotii
Source: 3 Biotech. 2014 Oct 31;5(5):647–51. doi: 10.1007/s13205-014-0262-5 (PMC4569624; doi:10.1007/s13205-014-0262-5)

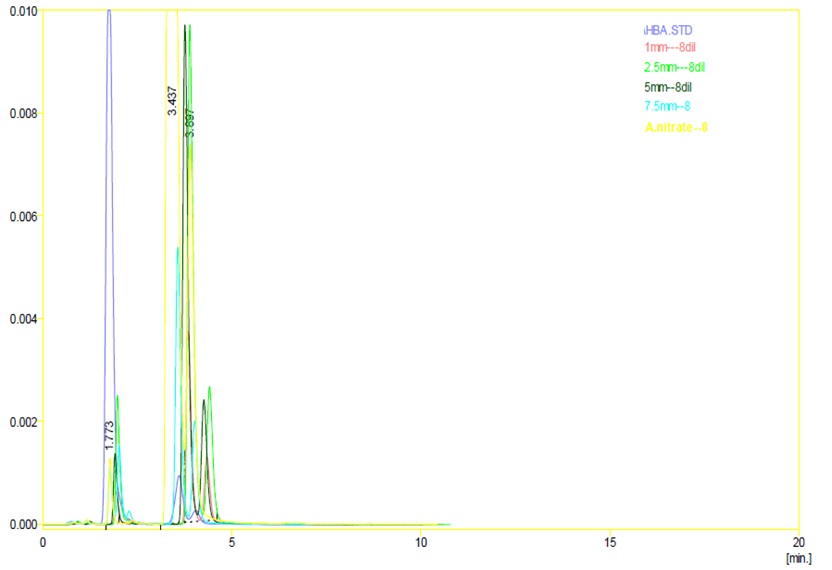

Supplement: Supplementary file 1 — Supplementary material 1 (JPEG 39 kb) [file 13205_2014_262_MOESM1_ESM.jpg]

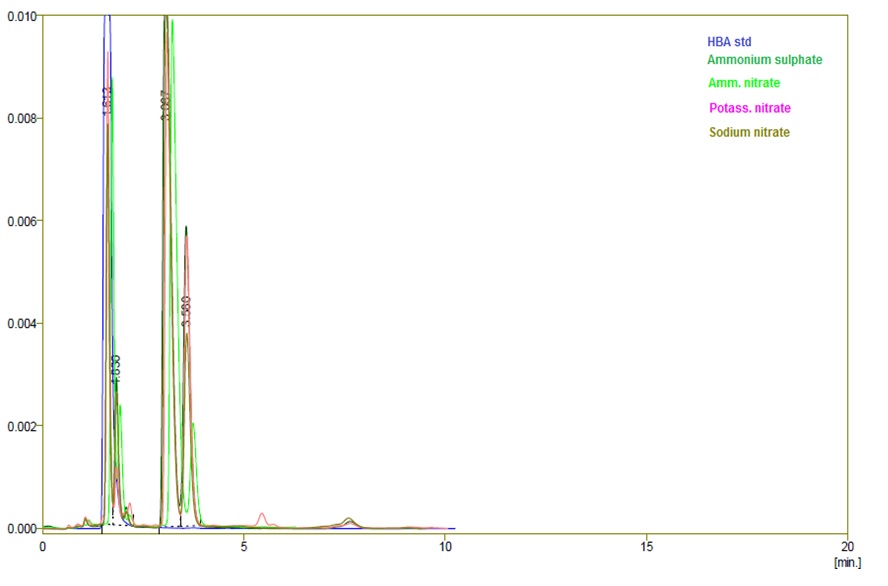

Supplement: Supplementary file 2 — Supplementary material 2 (JPEG 45 kb) [file 13205_2014_262_MOESM2_ESM.jpg]

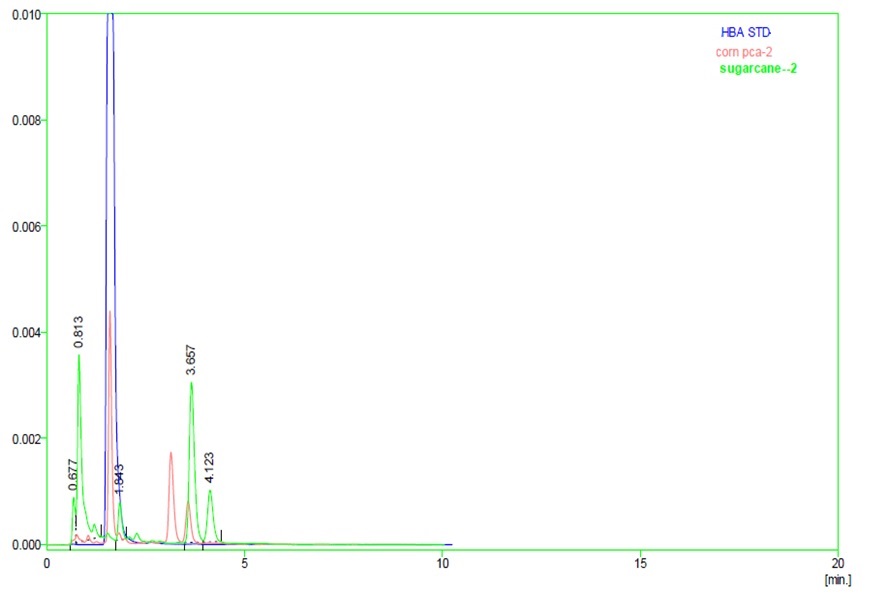

Supplement: Supplementary file 3 — Supplementary material 3 (JPEG 40 kb) [file 13205_2014_262_MOESM3_ESM.jpg]

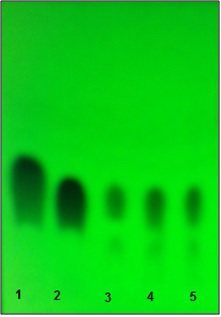

Supplement: Supplementary file 4 — Supplementary material 4 (JPEG 13 kb) [file 13205_2014_262_MOESM4_ESM.jpg]
