# Supplementary material for: Standardization of medium composition and agricultural waste in the production of p-hydroxybenzoic acid by Paecilomyces variotii
Source: 3 Biotech. 2014 Oct 31;5(5):647–51. doi: 10.1007/s13205-014-0262-5 (PMC4569624; doi:10.1007/s13205-014-0262-5)
Supplement: Supplementary file 5 — Supplementary material 5 (DOC 13 kb) [file 13205_2014_262_MOESM5_ESM.doc]

**Standardization of medium composition and agricultural waste in the production of *p-* hydroxybenzoic acid by *Paecilomyces variotii***

Jyothi Ramesh Jain^1^, Jimcy Thalakootoor John^1^, Ghosh Jyotirmoy^3^ and Shiragambi Hanmatagouda Manohar^1,2^*

^1^Department of Biotechnology, C.P.G.S. Jain University, 3^rd^ block, Jayanagar Bangalore 560011, India

^2^BCL, Biocon Research Limited, Biocon, Bangalore 560090, India

^3^National Institute of Animal Nutrition and Physiology, Adugodi, Bangalore 560030, India

* Author for correspondence:

E-mail: [manu2april@gmail.com](mailto:manu2april@gmail.com)

Ph : +91 9886530148

**Figure legends**

**Fig. S1** HPLC chromatogram of *Paecilomyces variotii* grown on varying concentration on the substrate (*p-*coumaric acid).

**Fig. S2** HPLC chromatogram of P. variotti grown on media supplemented with different nitrogen sources.

**Fig. S3** HPLC chromatogram of *P. variotti* grown on media supplemented with corn cob and sugarcane bagasse.

**Fig. S4** Thin layer chromatogram of culture filtrates exposed to UV light. Lane 1: Std *p-* coumaric acid; Lane 2 : Std *p-*hydroxybenzoic acid; Lane 3, 4 and 5 : processed culture filtrate (from day 2, day 4 and day 8)
